# Anatomical change of SMV branches after the Cattell Braasch maneuver facilitates safe resection around the uncinated process in pancreatoduodenectomy

**DOI:** 10.1186/s12893-021-01338-5

**Published:** 2021-09-08

**Authors:** Masayuki Akita, Eri Maeda, Tohru Nishimura, Koichiro Abe, Akihito Kozuki, Kunio Yokoyama, Tomohiro Tanaka, Shinji Kishi, Kunihiko Kaneda

**Affiliations:** Department of Surgery, Kakogawa Central City Hospital, Kakogawa, 675-8611 Japan

**Keywords:** Inferior pancreatoduodenal vein, Pancreatoduodenectomy, The Cattell Braasch maneuver

## Abstract

**Background:**

The aims of the present study were to demonstrate the anatomical change of superior mesenteric vein (SMV) branches and to show how the Cattell Braasch maneuver facilitates a safer ligation of these venous branches during a pancreatoduodenectomy (PD).

**Methods:**

Between January 2010 and December 2019, 97 patients with peripancreatic tumors underwent pancreatectomy. We retrospectively reviewed preoperative triple-phase enhanced computed tomography (CT) images and analyzed variations in SMV branches. Anatomical changes in SMV branches after the Cattell Braasch technique were observed using our operation video and illustrations.

**Results:**

The first jejunal vein (J1v) in 75% of patients ran posterior to the superior mesenteric artery (SMA), while the remainder (25%) ran anterior to it. The inferior pancreatoduodenal vein (IPDV) was preoperatively detected in 91% of patients. The IPDV drained into the J1v in 74% of patients and into the SMV in 37%. After the Cattell Braasch maneuver, the J1v which ran posterior to the SMA now was found to lie to the right anterolateral side the SMA and the visualization of both the J1v and the IPDV were much more clearly visualized.

**Conclusions:**

The most frequent venous variation was the IPDV draining into the J1v posterior to the SMA. After the Cattell Braasch maneuver, the IPDV was now located to the right anterolateral anterior aspect of the SMA which facilitates its visualization and should allow a safer ligation.

**Supplementary Information:**

The online version contains supplementary material available at 10.1186/s12893-021-01338-5.

## Background

In the past several decades, advances have been achieved in the techniques used for pancreatoduodenectomy (PD). Some artery-first approaches have recently been reported to improve surgical outcomes, particularly for pancreatic cancer [1–6]. Artery-first means the early ligation of branches of the superior mesenteric artery (SMA), such as the inferior pancreatoduodenal artery (IPDA) and jejunal arteries, which is useful for early decision-making regarding resectability during PD and reducing intraoperative blood loss [7, 8]. However, the contribution of these approaches to improvements in the short- and long-term survival of patients currently remains unclear [9, 10].

With the more widespread application of artery-first approaches, anatomical variations in SMA branches have been reported by gastrointestinal surgeons. And some studies have conducted detailed examinations on the anatomy of the superior mesenteric vein (SMV) and its branches [11–15]. Since tumors in pancreatic head cancer often invade the SMV with accompanying paraneoplastic inflammation, bleeding may occur during dissection between the uncinate process and SMV because of problems with good visualization of the first jejunal vein (J1v) and its anatomy. Surgeons are aware that this bleeding is caused by injury to the small vessels draining from the pancreatic head into the SMV or its jejunal branches. However, surgical ingenuity for the ligation of these veins has not yet been achieved. In our institutions, the Cattell Braasch maneuver has been applied to PD for the safe and complete resection of the mesopancreas [16, 17]. This artery-first approach mobilizes the right colon and small intestine, thereby maximizing the visualization of the venous branches and their common anomalies as they drain into the SMV and in their anatomic relationship to the SMA [16]. We also consider this method to be useful for avoiding injury to the small branches of the SMV by allowing better visualization of these venous branches.

The aims of the present study were to examine anatomical variations in SMV branches and demonstrate the advantage of the Cattell Braasch maneuver in PD.

## Methods

### Patient selection

The present study was approved by the Ethics Committee of Kakogawa Central City Hospital (#2019-90). Between January 2010 and December 2019, 97 patients with peripancreatic cancer underwent pancreatectomy at Kakogawa Central City Hospital; 95 with preoperative triple-phase computed tomography (CT) images (5-mm-thick slices) were enrolled in the present study.

### Imaging analysis

We retrospectively reviewed preoperative CT images of the study cohort, and assessed the following variations in J1v and inferior pancreatoduodenal vein (IPDV): whether the J1v ran anterior or posterior to the SMA, whether the IPDV was identical on CT images, and which veins the IPDV drained into.

Anatomically, the IPDV is divided into the anterior and posterior IPDV draining from the pancreatic head. However, we did not distinguish between these veins due to the difficulties associated with their identification on CT images. Patients with pancreatic cancer extensively involving the SMV and obliterating identification of the draining veins were excluded from the analysis of the IPDV (n = 9).

### The Cattell Braasch maneuver

Prior to the Cattell Braasch technique, Kocher’s maneuver was performed to identify the root of the left renal vein. Gastrectomy, lymph node dissection (stations 8, 9, and 12), bile duct resection upstream of the confluence of the cystic duct, and ligation of the gastroduodenal artery (GDA) were then completed.

Mobilization and resection of the pancreatic head and uncinate process from the SMV and the SMA proceeds as follows. (Additional file 1: Supplemental video).

First, a Cattell Braasch maneuver is performed as follows.

**Fig. 1:**
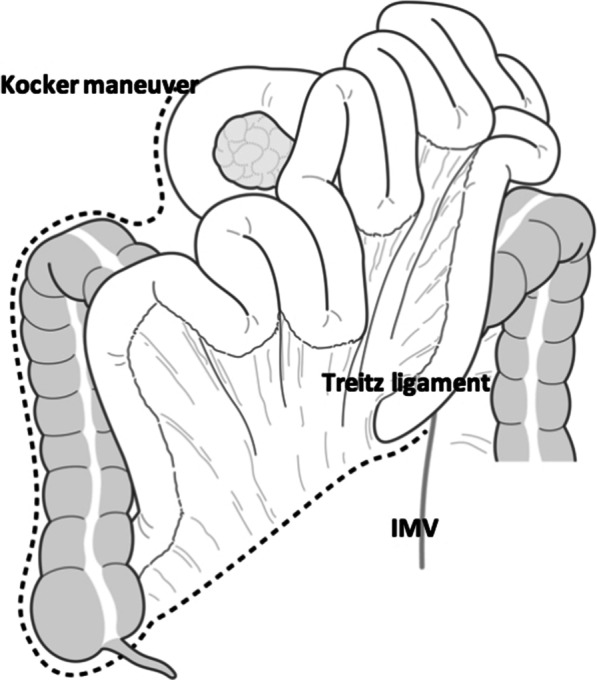
The Cattell Braasch maneuver consists of mobilization of the right colon, cecum, and small intestine from a retroperitoneal plane with transection of the Treitz ligament. *IMV* inferior mesenteric vein. (The authors own the copyright of this image.)

1. Mobilization of the right colon, cecum, and mesenteric root of the small intestine from a retroperitoneal plane with transection of the Treitz ligament (Fig. 1).
2. De-rotation of the duodenum and mobilized small intestine/right colon to resemble the positions observed in those with intestinal malrotation, i.e. clockwise reduction of the small intestine around the SMA and SMV. Thereby clarifying and facilitating the identification of the relationship of the SMA and SMV at the base of the small bowel mesentery.

Next, the mobilization of the duodenum, proximal jejunum and pancreatic head from the SMV then proceeds as follows.

3. Transection of the jejunum between the 1st and 2nd branches off the SMA.
4. Resection of the jejunal mesentery toward the SMA. Ligation of the IPVDs which after the Cattell Braasch maneuver now lie more anterolateral from where they had originally coursed posterior or anterior to the SMA and are much more easily visualized, because the small bowel and right colon are retracted anteriorly by the full Cattell Braasch maneuver.
5. Resection of the uncinate process and right posterior aspect of the SMA from the back side of the pancreas head along the SMA, followed by ligation of the origin of the IPDA.
6. Resection of the uncinate process and SMV from the dorsal side of the pancreas head and the ligation of its branches (including the gastrocolic trunk and/or the IPDV directly draining into the SMV) from the uncinate process.
7. Before reconstruction, restoration of the small bowels and right colon to its normal anatomic position without any attempt to fixate the total of the derotated bowel.

## Results

Between 2010 and 2019, 97 patients with peripancreatic cancer underwent pancreatectomy in our hospital: 92 PD and 5 total pancreatectomy. Seventy-two patients were diagnosed with pancreatic cancer, 14 with distal bile duct cancer, 10 with cancer of the duodenal papilla, and 1 with duodenal cancer.

**Fig. 2:**
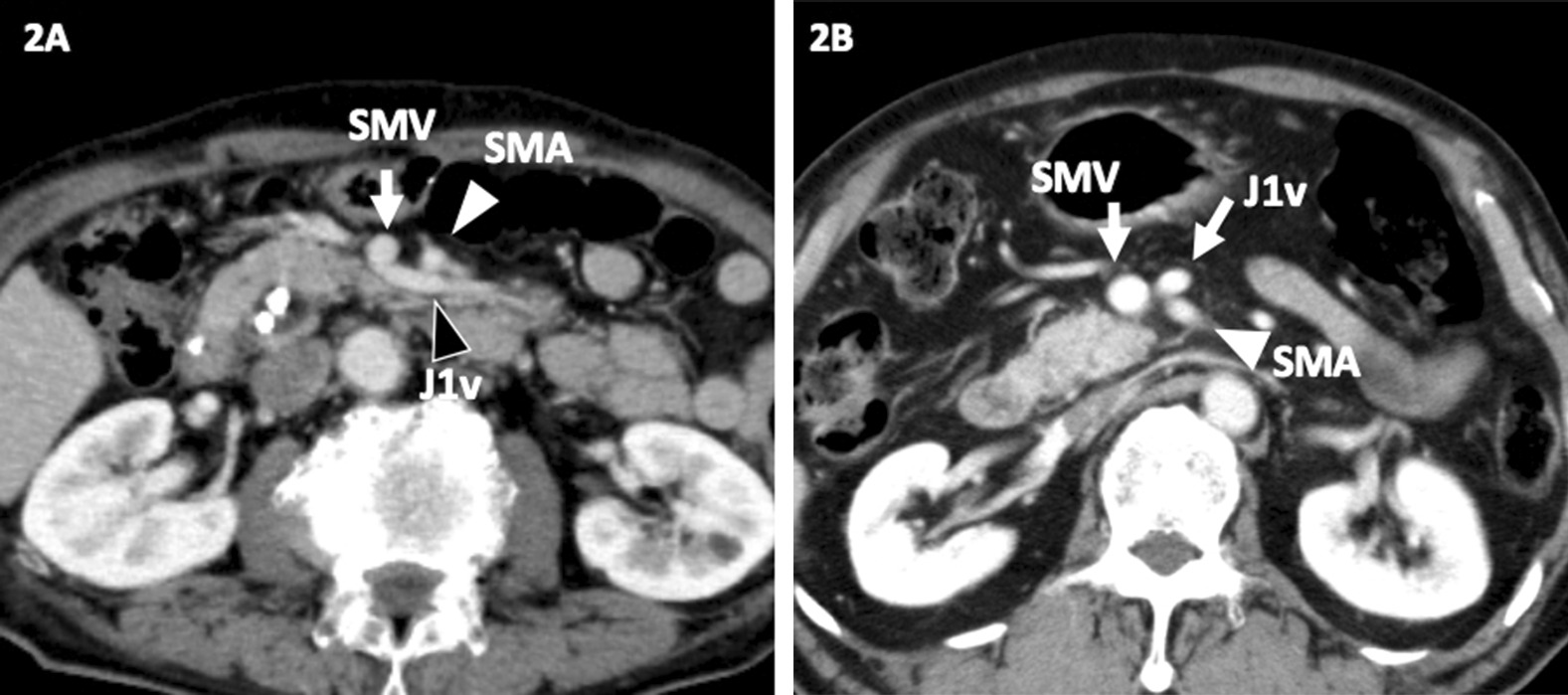
**A**, **B** Variations in the first jejunal vein (J1v). The J1v drained into the SMV from posterior (**A**) and anterior (**B**)

The imaging analysis showed that the J1v in 71 patients (75%) ran posterior to the SMA (Fig. 2A), whereas it ran anterior to it in 24 (25%) (Fig. 2B). Among the J1v running posterior to the SMA, 70% (50/71) drained into the caudal side of a confluence of the gastro-colic trunk (GCT), while 88% (21/24) running anterior to the SMA drained into the cranial side of the confluence.

**Fig. 3:**
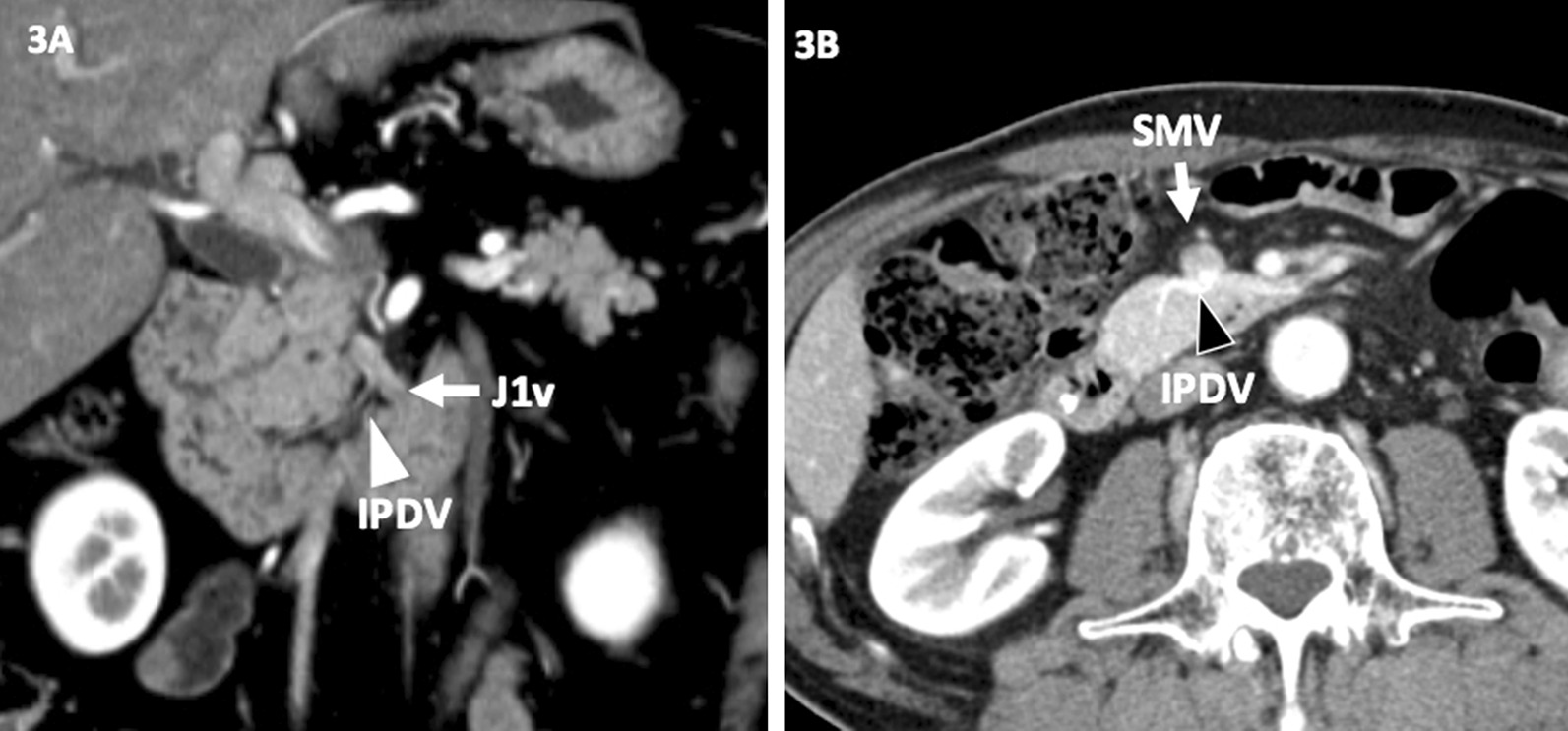
**A**, **B** Variations in the inferior pancreatoduodenal vein (IPDV). The IPDV drained into the J1v (**A**) and SMV (**B**)

The IPDV(s) was detected on CT images in 78 patients (91%, 78/86). Of these, the IPDVs in 9 patients drained into both the J1v and SMV. The IPDV drained into the J1v in 58 patients (Fig. 3A) and into the SMV in 29 (Fig. 3B). The majority of IPDV directly draining into the SMV (83%) fed into the posterior aspect of the SMV.

In 54 patients with the IPDV draining into the J1v, the J1v ran posterior to the SMA (54/58, 93%). Among those with the IPDV directly draining into the SMV, the J1v ran anterior to the SMA (14/29, 48%).

## Discussion

PD may be safely performed due to the accumulation of information on the peri-pancreatic anatomy and development of surgical techniques and devices. One of the current surgical trends in pancreatic cancer is the artery-first approach, with surgeons suggesting several different methods with surgical benefits [1–6]. Therefore, anatomical variations in the SMA and its branches are now well known and surgeons routinely assess arterial anomalies at the same time as tumor staging and its resectability on preoperative images when preparing for PD.

**Fig. 4:**
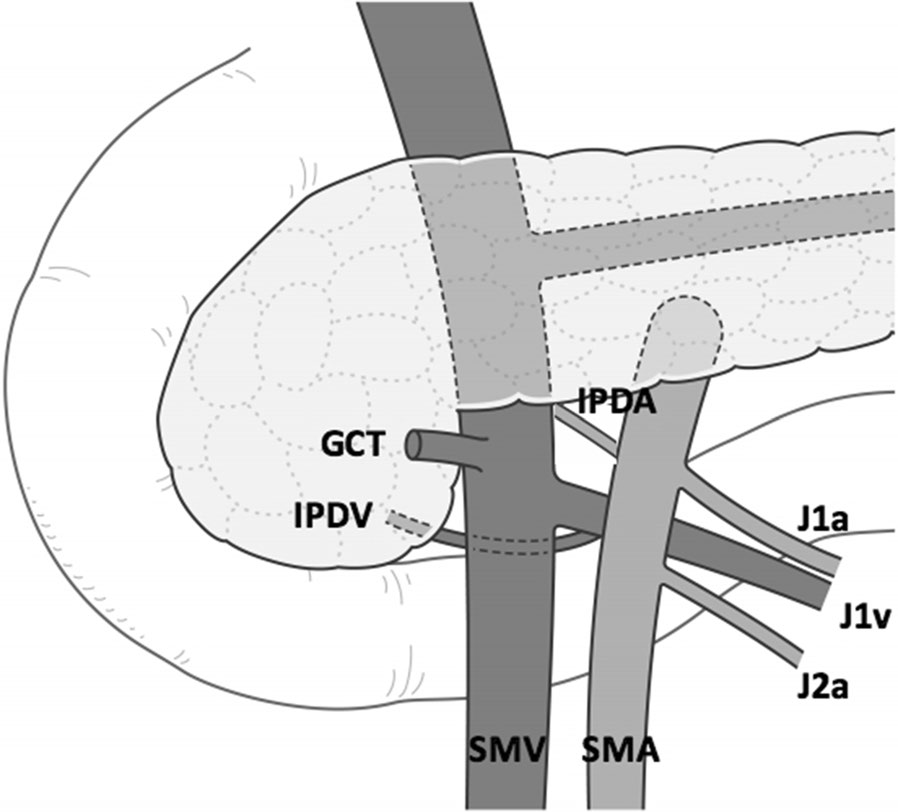
The most common variation of SMV branches. The IPDV drained into the J1v running posterior the SMA. *GCT* gastrocolic trunk, *IPDV* inferior pancreatoduodenal vein, *J1a/J2a* first and second jejunal artery, *J1v* first jejunal vein, *IPDA* inferior pancreatoduodenal artery. (The authors own the copyright of this image.)

The venous anatomy around the pancreas also has gradually been clarified [11–15]. Surgeons sometimes encounter unanticipated bleeding, particularly during dissection between the uncinate process and SMV and IPDA, which may be caused by unintentional injury to the IPDV located posterior to the SMV and SMA. The following variations have been reported in SMV branches. The J1v crossed the SMA anteriorly in 17.0–32.4% cases [18–21]. Although the incidence of the IPDV on images has not been reported, the IPDV was identified in more than 75% of cases in previous studies using cadavers [12, 14]. Another study showed that the IPDV commonly drained into the J1v on CT images (93%) [13]. These findings are consistent with the present results, which showed that the most common variation was the J1v running posterior to the SMA with the IPDV draining into the J1v (Fig. 4). We also found that the J1v crossing the SMA anteriorly frequently drained into the cranial side of the GCT confluence, while the IPDV fed into the posterior aspect of the SMV in the majority of patients (83%). However, to the best of our knowledge, there has only been one report on the practical relevance of these results [16].

**Fig. 5:**
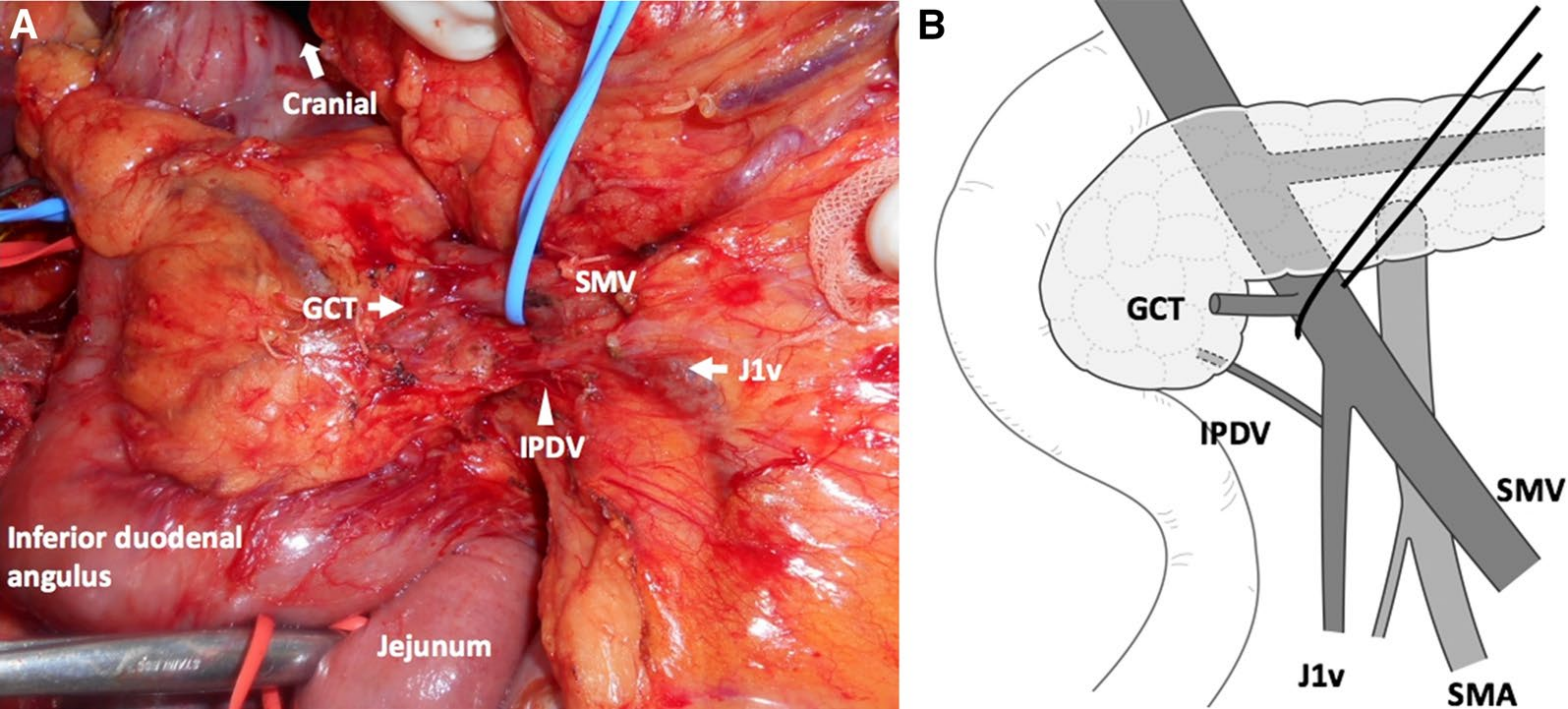
After the Cattell Braasch maneuver, the J1v and IPDV moved above the SMA. The blue vessel loop was around the SMV. The SMA lied left and posterior to the SMV. *GCT* gastrocolic trunk, *IPDV* inferior pancreatoduodenal vein, *J1v* first jejunal vein. (The authors own the copyright of this image.)

The Cattell Braasch maneuver was initially introduced as a method for exposing the third and fourth portions of the duodenum by derotating all of the small intestine and right colon [17]. We previously reported the usefulness of the Cattell Braasch maneuver as an artery-first approach for PD [22]. It facilitates safe PD by simplifying the torsional position between the duodenum and SMA/SMV. In terms of venous variations, after the elimination of intestinal rotation by the Cattell Braasch maneuver, the J1v running posterior to the SMA appeared at the right anterolateral aspect of the SMA, and the IPDV draining into the J1v was clearly visualized (the most common variation, Fig. 5A, B). In patients with the IPDV draining directly into the SMV, which frequently feeds into the posterior aspect of the SMV and is difficult to identify under a direct view, the posterior aspect of the SMV was rotated to the right anteroposterior aspect of the SMV and the IPDV was safely ligated.

Anatomical changes in SMV branches are not the main reason for performing the Cattell Braasch maneuver. However, the concept of this maneuver will facilitate the avoidance of uncontrollable bleeding during dissection around the SMV. We would like to emphasize that this technique can also be appropriate for the neoplasm arising from the uncinate process with inflammation around the SMV, such as intraductal papillary mucinous neoplasm. And pancreatobiliary surgeons need to be aware of this technique as a surgical option.

There are some limitations that need to be addressed. This study was retrospective and a “How I Do it” report. We performed the Cattell Braasch maneuver mainly for pancreatic ductal adenocarcinoma with SMV invasion and standard PD for other peripancreatic cancers. Therefore, the reductive impact of the Cattell Braasch maneuver on intraoperative bleeding currently remains unclear.

## Conclusion

The Cattell Braasch maneuver simplifies the anatomy of SMV branches and is useful for the safe ligation of these vessels.

## Supplementary Information

**Additional file 1: Video S1.** The procedure of theCattell Braasch maneuver. The Cattell Braasch maneuver is composed ofmobilization of the right hemi-colon and the total small intestine. De-rotatingthese bowels facilitates safe mesopancreas resection.

## Data Availability

Data that support the results of the present study are available upon reasonable request from the corresponding author.

## Abbreviations

SMV: Superior mesenteric vein
PD: Pancreatoduodenectomy
CT: Computed tomography
J1v: First jejunal vein
SMA: Superior mesenteric artery
IPDV: Inferior pancreatoduodenal vein
IPDA: Inferior pancreatoduodenal artery
GDA: Gastroduodenal artery
GCT: Gastro-colic trunk
